# Identification of Uncommon *Cryptosporidium* *viatorum* (a Novel Subtype XVcA2G1c) and *Cryptosporidium andersoni* as Well as Common *Giardia duodenalis* Assemblages A and B in Humans in Myanmar

**DOI:** 10.3389/fcimb.2020.614053

**Published:** 2020-11-25

**Authors:** Yanchen Wu, Baiyan Gong, Xiaohua Liu, Yanyan Jiang, Jianping Cao, Lan Yao, He Li, Aiqin Liu, Yujuan Shen

**Affiliations:** ^1^ Department of Parasitology, Harbin Medical University, Harbin, China; ^2^ National Institute of Parasitic Diseases, Chinese Center for Disease Control and Prevention, Chinese Center for Tropical Diseases Research, WHO Collaborating Centre for Tropical Diseases, National Center for International Research on Tropical Diseases, Ministry of Science and Technology, Key Laboratory of Parasite and Vector Biology, MOH, Shanghai, China

**Keywords:** *Cryptosporidium*, *Giardia*, humans, genotyping, subtyping

## Abstract

*Cryptosporidium* and *Giardia* are two important zoonotic intestinal protozoa responsible for diarrheal diseases in humans and animals worldwide. Feces from infected hosts, water and food contaminated by *Cryptosporidium* oocysts and *Giardia* cysts as well as predictors such as poverty have been involved in their transmission. Myanmar is one of the world’s most impoverished countries. To date, there are few epidemiological studies of *Cryptosporidium* and *Giardia* in humans. To understand the prevalence and genetic characterization of *Cryptosporidium* spp. and *Giardia duodenalis* in humans in Myanmar, a molecular epidemiological investigation of the two protozoa was conducted in four villages of Shan State. 172 fecal specimens were collected from Wa people (one each) and identified for the presence of *Cryptosporidium* spp. and *G. duodenalis* by sequence analysis of their respective small subunit ribosomal RNA genes. 1.74% of investigated people were infected with *Cryptosporidium* spp.—*C. andersoni* (n = 2) and *C. viatorum* (n = 1) while 11.05% infected with *G. duodenalis*—assemblages A (n = 6) and B (n = 13). By sequence analysis of 60-kDa glycoprotein gene, the *C. viatorum* isolate belonged to a novel subtype XVcA2G1c. DNA preparations positive for *G. duodenalis* were further subtyped. Five of them were amplified and sequenced successfully: different assemblage B sequences (n = 2) at the triosephosphate isomerase (tpi) locus; sub-assemblage AII sequence (n = 1) and identical assemblage B sequences (n = 2) at the β-giardin (bg) locus. This is the first molecular epidemiological study of *Cryptosporidium* spp. and *G. duodenalis* in humans in Myanmar at both genotype and subtype levels. Due to unclear transmission patterns and dynamics of *Cryptosporidium* spp. and *G. duodenalis*, future research effort should focus on molecular epidemiological investigations of the two parasites in humans and animals living in close contact in the investigated areas, even in whole Myanmar. These data will aid in making efficient control strategies to intervene with and prevent occurrence of both diseases.

## Introduction

*Cryptosporidium* and *Giardia* are two ubiquitous intestinal protozoan parasites in humans and numerous animals. Both cryptosporidiosis and giardiasis are clinically characterized by diarrhea, and the severity of diarrhea is closely related to the age and health status of the infected hosts as well as the genetic background and infective dose of the parasites (Xiao and Fayer, 2008). Immunocompetent individuals typically experience self-limiting diarrhea and are often asymptomatic while chronic diarrhea in immunocompromised individuals (Plutzer et al., 2018). Severe life-threatening diarrhea has been reported in cryptosporidiosis patients infected with human immunodeficiency virus (HIV) (Ryan et al., 2016). The infective dose of the two parasites are low: <10 *Cryptosporidium* (*Cryptosporidium hominis* or *Cryptosporidium parvum*) oocysts and 10–100 *Giardia duodenalis* (syn. *Giardia intestinalis*, *Giardia lamblia*) cysts can cause infection in immunocompetent persons (Rendtorff, 1954; Okhuysen et al., 1999; Chappell et al., 2006). More seriously, a single *C. parvum* oocyst has been reported to initiate infection in immunosuppressed persons (Zhao et al., 2014). In general, the pathogenicity of *Cryptosporidium* is considered to be more severe in humans than that of *Giardia* (Prado et al., 2005). Humans can acquire *Cryptosporidium* and *Giardia* infections through the fecal-oral route, either directly (via human-to-human/animal contact) or indirectly (via ingestion of contaminated water or food) (Osman et al., 2016). The role of water and food in the epidemiology of the two parasitic diseases is now well recognized. To date, waterborne and foodborne outbreaks of cryptosporidiosis (>524 and >26) (Liu et al., 2020) and giardiasis (>344 and >38) had been reported worldwide (Karanis et al., 2007; Baldursson and Karanis, 2011; Efstratiou et al., 2017; Ryan et al., 2019). Based on clinical and public health importance, *Cryptosporidium* and *Giardia* are listed on the Environmental Protection Agency (EPA) microbial contaminant candidate list of concern for waterborne transmission (https://www.epa.gov/ground-water-and-drinking-water/national-primary-drinking-water-regulations). The two pathogens have also been ranked as the 5th and 11th most important foodborne parasites worldwide by a joint Food and Agriculture Organization (FAO)/World Health Organization (WHO) in 2014, respectively (Plutzer et al., 2018).

Both *Cryptosporidium* and *Giardia* are complicated genera. To date, 41 *Cryptosporidium* species and over 40 genotypes have been recognized (Feng et al., 2018; Holubová et al., 2019; Bolland et al., 2020; Holubová et al., 2020). Among them, 22 *Cryptosporidium* species/genotypes have been identified in humans (Xiao and Feng, 2017; Kváč et al., 2018), and *C. hominis* and *C. parvum* are the two most common species, reported in > 90% of human cryptosporidiosis cases (Squire and Ryan, 2017). However, in some countries, especially in developing countries, some unusual species have a high occurrence in human cryptosporididosis cases, such as *Cryptosporidium meleagridis* (10–20%) in Thailand and in Peru (Gatei et al., 2002; Cama et al., 2007; Cama et al., 2008); *Cryptosporidium andersoni* (79.59%) in India (Hussain et al., 2017); *Cryptosporidium viatorum* (7.14–11.11%) in Ethiopia (Adamu et al., 2014; de Lucio et al., 2016). Among the eight recognized species, and only *G. duodenalis* has been found to infect humans with eight assemblages (A to H) being identified (Ryan et al., 2019). Assemblages A and B are responsible for the vast majority (99%) of human giardiasis cases, and both of them have also been found in a variety of mammal species (Sprong et al., 2009). Assemblages C to H are specific to some animal species, but assemblages C to F are occasionally found in humans (Cacciò et al., 2018).

Cryptosporidiosis and giardiasis cause considerable human disease burdens worldwide. Like other infectious diseases, these two parasitic diseases also usually affect people living in poverty, and may further promote poverty. Developing countries usually have higher prevalence than developed countries: 5–10% versus 1% for cryptosporidiosis (Checkley et al., 2015); 0.9–40.7% versus 0.4–7.0% for giardiasis (Feng and Xiao, 2011; Ryan and Cacciò, 2013). In fact, early in 2004, *Cryptosporidium* spp. and *G. duodenalis* were included in the WHO’s “Neglected Disease Initiative” due to their link with poverty (Savioli et al., 2006). Myanmar is one of the world’s most impoverished countries. As of 2019, Myanmar ranks 145 out of 189 countries according to the Human Development Index (http://hdr.undp.org/en/content/2019-human-development-index-ranking). However, it is unclear on epidemiological status of human cryptosporidiosis and giardiasis and genetic characterization of *Cryptosporidium* spp. and *G. duodenalis*. To date, only two studies reported the prevalence by microscopy in Myanmar: *Cryptosporidium* spp. (3.4%, 7/203) in infants with diarrhea (Aye et al., 1994) and *G. duodenalis* (3.4%, 28/821) in schoolchildren and guardians (Kim et al., 2016). The present study was conducted to understand the prevalence and genetic characterization of *Cryptosporidium* spp. and *G. duodenalis* in humans in Myanmar at the genotype and subtype levels.

## Materials and Methods

### Ethics Statement

Scientific approval and ethical clearance for this study was given by the Ethics Committee of the National Institute of Parasitic Diseases, Chinese Center for Disease Control and Prevention, China, and the Myanmar Eastern Shan State Special Region 2 Ethic Health Organization. All study participants were informed of the aims and the procedures of this study at enrollment. Before collection of fecal specimens, written informed consents for all study participants were obtained from each adult individual or legal guardian of enrolled children.

### Study Site and Collection of Fecal Specimens

**Figure 1:**
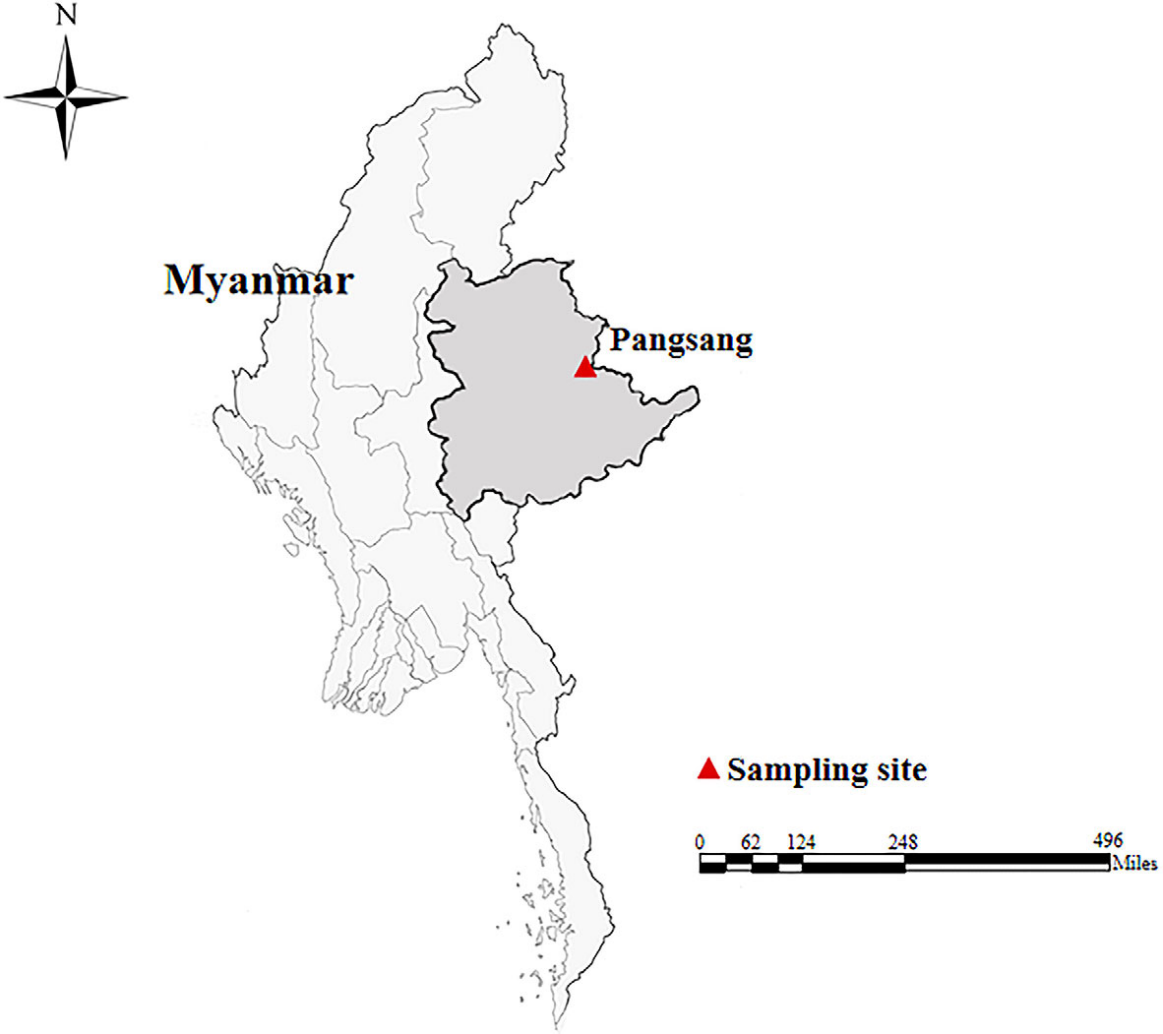
A map showing the sampling site of human fecal specimens in this study.

In October, 2018, a total of 172 fecal specimens (approximately 5–10 g each) were collected from Wa people (one specimen each) in four villages of Pangsang Township (22˚10′N, 99˚11′E) of Matman District of Shan State, which is located in the east of Myanmar, bordering with China’s Yunnan Province in about 133-kilometer-long border line (**Figure 1**). The villages investigated have poor sanitation and garbage collection. Domestic pigs and chickens are the most common animals, which are kept by almost every household. The villagers are poorly educated and have weak hygiene awareness. The participants were composed of children (n = 97), teenagers (n = 41) and adults (n = 34), with their ages ranging from seven to 53 years. At the time of sampling, we only recorded the presence or absence of diarrhea. All fecal specimens were delivered to the laboratory in a cooler with ice packs within 24 h after collection and stored in a refrigerator at −20°C for future analysis.

### DNA Extraction

Genomic DNA was extracted directly from approximately 180 to 200 mg of each fecal specimen using a QIAamp DNA Mini Stool Kit (Qiagen, Hilden, Germany) according to the manufacturer-recommended procedures. To obtain a high yield of DNA, the lysis temperature was increased to 95°C. DNA was eluted in 200 μl of AE elution buffer and stored at −20°C. Extracted DNA preparations were analyzed by nested polymerase chain reaction (PCR) amplification.

### Genotyping and Subtyping of *Cryptosporidium* spp. and *G. duodenalis*

*Cryptosporidium* species were identified by nested PCR amplification of the partial small subunit ribosomal RNA (SSU rRNA) gene (approximately 830 bp) (Huang et al., 2016). Subtyping of DNA preparations positive for *Cryptosporidium* at the SSU rRNA locus was performed by nested PCR amplification of the partial 60 kDa glycoprotein (gp60) gene (approximately 950 bp) (Stensvold et al., 2015). Meanwhile, all DNA preparations were screened for the presence of *G. duodenalis* by nested PCR amplification of the partial SSU rRNA gene (approximately 290 bp) and were identified to the assemblage level as previously described by Appelbee et al. (2003). DNA preparations positive for *G. duodenalis* at the SSU rRNA locus were further analyzed to determine sub-assemblages by nested PCR amplification of the triose phosphate isomerase (tpi) and β-giardin (bg) genes. Assemblage A/B-specific nested PCRs were performed to amplify approximately 330 and 460 bp nucleotide fragments of the tpi gene, respectively (Geurden et al., 2008; Levecke et al., 2009). Approximately 510 bp fragment of bg gene was amplified (Lalle et al., 2005).

Each DNA preparation was performed two times and TaKaRa Taq DNA polymerase (TaKaRa Bio Inc., Tokyo, Japan) was used for all PCR reactions. A negative control (DNase-free water) and a positive control (*C. baileyi* or *G. duodenalis* assemblage E) were included in all PCR tests. All secondary PCR products were subjected to electrophoresis in a 1.5% agarose gel and visualized by staining the gel with GelStrain (TransGen Biotech., Beijing, China) before sequencing.

### Sequence Analysis

Positive secondary PCR products of expected size were sent to Comate Bioscience Company Limited (Jilin, China) for sequencing using their respective secondary PCR primers on an ABI PRISM 3730 XL DNA Analyzer using the BigDye Terminator v3.1Cycle Sequencing Kit (Applied Biosystems, Carlsbad, CA, USA). The accuracy of the sequencing data was confirmed by sequencing in both directions. Species/genotypes and subtypes of *Cryptosporidium*, and assemblages and sub-assemblages of *G. duodenalis* were identified by comparing the nucleotide sequences obtained in the present study with reference sequences downloaded from GenBank using the Basic Local Alignment Search Tool (BLAST) (http://www.ncbi.nlm.nih.gov/blast/) and Clustal X 1.83 (http://www.clustal.org/).

### Phylogenetic Analysis

To assess phylogenetic relationships among *C. viatorum* subtypes obtained in the present study and those published in GenBank databases, all gp60 gene sequences of *C. viatorum* subtypes were implemented in the software Mega 5 (http://www.megasoftware.net/). A neighbor-joining tree was constructed based on the evolutionary distances calculated by the Kimura 2-parameter model. The reliability of the trees was assessed using the bootstrap analysis with 1,000 replicates.

### Statistical Analysis

All statistical analyses were performed with Statistical Package for the Social Sciences (SPSS) 19.0. Pearson chi-square (χ^2^) and Fisher’s exact tests were used to determine statistical significance in the present study. All results were interpreted using odds ratios, 95% confidence intervals and significance level (*P*-values < 0.05).

### Nucleotide Sequence Accession Numbers

The representative nucleotide sequences obtained in this study were deposited in the GenBank database under the following accession numbers: MW014313 to MW014315 (SSU rRNA) and MW014316 (gp60) for *Cryptosporidium*; MW011715 and MW011716 (tpi), MW011717 and MW011718 (bg) for *G. duodenalis*.

## Results

### Prevalence of *Cryptosporidium* spp. and *G. duodenalis*

All 172 fecal specimens were screened for the presence of *Cryptosporidium* spp. and *G. duodenalis* by PCR amplification and sequence analysis of their respective partial SSU rRNA gene. *G. duodenalis* (11.05%, 19/172) was observed to be more prevalent than *Cryptosporidium* spp. (1.74%, 3/172) in the investigated people. *Cryptosporidium* spp. was found only in children (1.03%, 1/97) and adults (5.88%, 2/34) while *G. duodenalis* only in children (12.37%, 12/97) and teenagers (17.07%, 7/41) (**Table 1**).

**Table 1:** Prevalence and genetic characterization of *Cryptosporidium* spp. and *G. duodenalis* in humans.

Age group (years)	Examined no.	*Cryptosporidium* spp.			*G. duodenalis*			
		No. of positive (%)	Species	Subtype	No. of positive (%)	Assemblage	Sub-assemblage	
			SSU rRNA (n)	Gp60 (n)		SSU rRNA (n)	tpi (n)	bg (n)
Children (<13)	97	1 (1.03)	*C. viatorum* (1)	XVcA2G1c (1)	12 (12.37)	B (9); A (3)	B-I (1)	B (2); AII (1)
Teenagers (13–17)	41	0	−	−	7 (17.07)	B (4); A (3)	B-II (1)	−
Adults (≥18)	34	2 (5.88)	*C. andersoni* (2)	−	0	−	−	−
Total	172	3 (1.74)	*C. viatorum* (1); *C. andersoni* (2)	XVcA2G1c (1)	19 (11.05)	B (13); A(6)	B-I (1); B-II (1)	B (2); AII (1)

The bars denote negative results at the locus.

By χ^2^ tests, only a statistically higher prevalence of *G. duodenalis* was observed in children than in adults (*P* = 0.04). Both prevalences of *Cryptosporidium* spp. and *G. duodenalis* were higher in people without diarrhea than those with diarrhea (**Table 2**). Furthermore, there were no relationships between *Cryptosporidium* spp. or *G. duodenalis* infection and diarrhea in each age group (*P* > 0.05) (**Table 3**).

**Table 2:** Prevalence of *Cryptosporidium* spp. and *G. duodenalis* by age and symptom.

Group		Examined no.	*Cryptosporidium* spp.			*G. duodenalis*		
			Positive no. (%)	OR^a^ (95% CI^b^)	*χ* ^2^/*P*-value	Positive no. (%)	OR^a^ (95% CI^b^)	*χ* ^2^/*P*-value
Age	Children	97	1 (1.03)	Ref		12 (12.37)	Ref	
	Teenagers	41	0	0.99 (0.97, 1.01)	−/1.00^c^	7 (17.07)	0.69 (0.25, 1.89)	0.54/0.46
	Adults	34	2 (5.88)	0.17 (0.02, 1.90)	0.92/0.34	0	0.88 (0.81, 0.94)	−**/0.04** ^c,d^
Symptom	Diarrhea	42	0	1.02 (1.00, 1.05)	−/1.00^c^	4 (9.52)	0.81 (0.25, 2.58)	0.01/0.94
	Non-diarrhea	130	3 (2.31)			15 (11.54)		

^a^OR Odds ratio. ^b^CI Confidence interval. ^c^Fisher’s exact test. ^d^Bold type for values indicates statistical significance.

**Table 3:** Relationships between *Cryptosporidium* spp. or *G. duodenalis* infection and diarrhea in each age group.

Group	Symptom	Examined no.	*Cryptosporidium* spp.			*G. duodenalis*		
			Positive no. (%)	OR^a^ (95% CI^b^)	*χ* ^2^/*P*-value	Positive no. (%)	OR^a^ (95% CI^b^)	*χ* ^2^/*P*-value
Children	Diarrhea	26	0	1.01 (0.99, 1.04)	−/1.00^c^	2 (7.69)	0.51 (0.10, 2.49)	0.25/0.62
	Non-diarrhea	71	1 (1.41)			10 (14.08)		
Teenagers	Diarrhea	14	0	−	−	2 (14.29)	0.73 (0.12, 4.37)	−/1.00
	Non-diarrhea	27	0			5 (18.52)		
Adults	Diarrhea	2	0	1.07 (0.96, 1.17)	−/1.00^c^	0	−	−
	Non-diarrhea	32	2 (6.25)			0		

^a^OR Odds ratio, ^b^CI Confidence interval, ^c^Fisher’s exact test.

### Genotyping and Subtyping of *Cryptosporidium*

Sequence analysis of the SSU rRNA gene identified two *Cryptosporidium* species: *C. andersoni* (n = 2) and *C. viatorum* (n = 1). Two *C. andersoni* isolates (MW014313 and MW014314) had 99.73% (two-base difference) and 99.62% (three-base difference) similarity with *C. andersoni* isolates from a dairy calf (JX515549), respectively. *C. viatorum* isolate was identical to a wild rat-derived *C. viatorum* isolate (MK522269).

**Figure 2:**
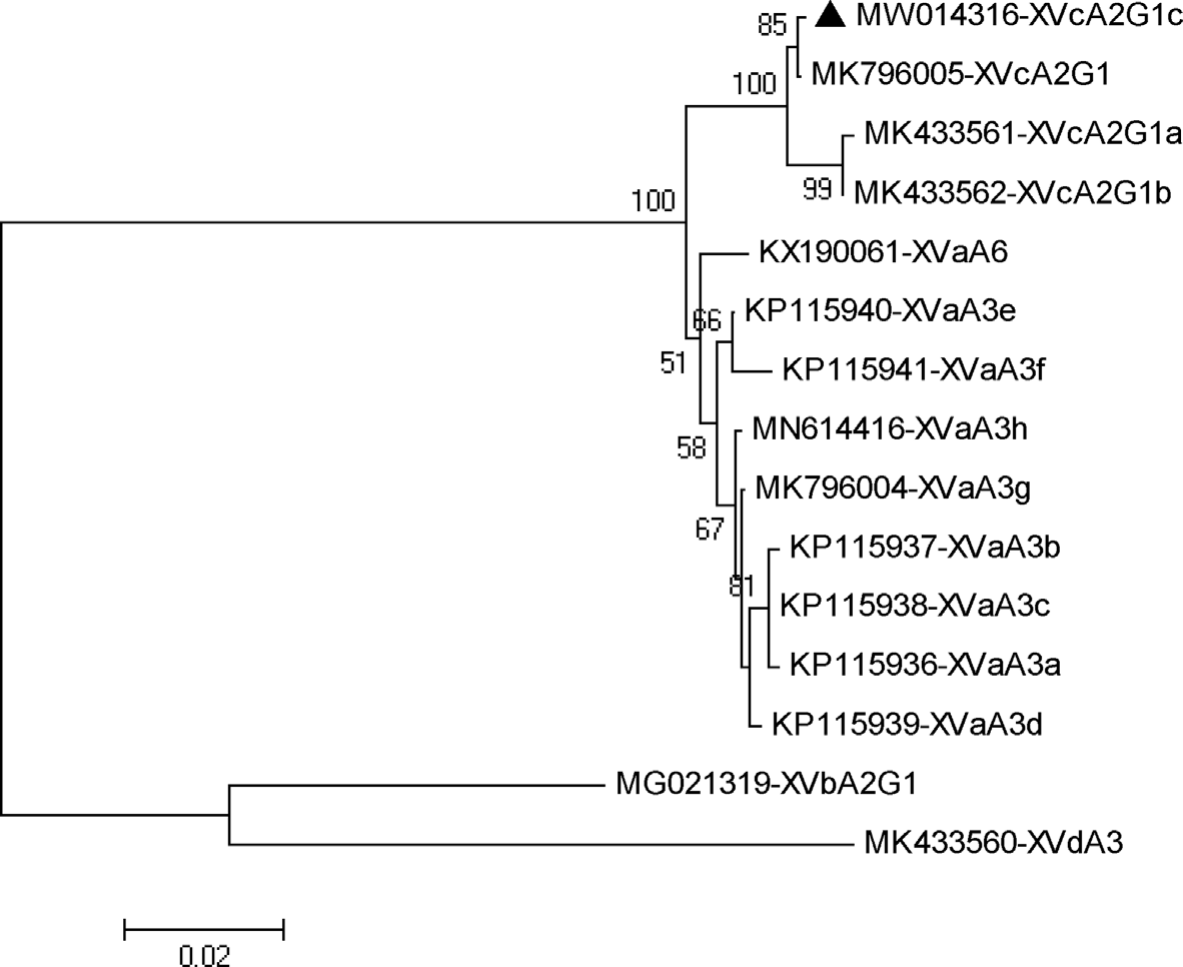
Phylogenetic relationship of gp60 subtypes of *Cryptosporidium viatorum*. The relationships among *C. viatorum* subtypes identified in the present study and those deposited in the GenBank were inferred by a neighbor-joining analysis of gp60 gene sequences based on genetic distance by the Kimura 2-parameter model. The numbers on the branches are percent bootstrapping values from 1000 replicates. Each sequence is identified by its accession number and subtype designation. The triangle filled in black indicates the subtype identified in this study.

In phylogenetic analysis of the gp60 gene sequences, the *C. viatorum* isolate obtained in the present study was grouped with *C. viatorum* subtypes XVcA2G1b, XVcA2G1a, and XVcA2G1 (**Figure 2**). Meanwhile, this result was also supported by evolutionary analysis at the nucleotide and amino acid levels: low genetic variations (0.12–0.99% and 0.39–1.96%) compared to the other three subtypes in XVc subtype family; high genetic variations (2.13–21.47% and 3.59–35.60%) compared to all 11 subtypes in subtype families (XVa, XVb, XVd) (**Table 4**). According to the terminology of *C. viatorum* subtypes established by Stensvold et al. (2015), a novel subtype XVcA2G1c was identified, which had the largest similarity (99.88%) with subtype XVcA2G1 (MK796005) from a Bower’s white-toothed rat in China.

**Table 4:** Pairwise differences (percentage) among gp60 subtypes of *C. viatorum* for nucleotide (below the diagonal) and amino acid sequences (above the diagonal).

	1	2	3	4	5	6	7	8	9	10	11	12	13	14	15		
1	–	0	0	0.79	0.79	2.38	0.40	0.79	3.16	28.40	4.40	5.20	5.20	4.40	33.20	1	XVaA3a
2	0.25	–	0	0.79	0.79	2.38	0.40	0.79	3.16	28.40	4.40	5.20	5.20	4.40	33.20	2	XVaA3b
3	0.12	0.12	–	0.79	0.79	2.38	0.40	0.79	3.16	28.40	4.40	5.20	5.20	4.40	33.20	3	XVaA3c
4	0.50	0.50	0.37	–	0.79	2.39	0.40	0.79	3.17	28.92	4.42	5.22	5.22	4.42	34.14	4	XVaA3d
5	1.00	1.00	0.87	0.75	–	1.58	0.39	0	2.75	28.97	3.97	4.76	4.76	3.97	33.73	5	XVaA3e
6	1.50	1.50	1.38	1.25	0.49	–	1.98	1.58	2.77	29.60	4.00	4.80	4.80	4.00	33.60	6	XVaA3f
7	0.49	0.49	0.37	0.25	0.62	1.12	–	0.39	2.76	29.08	3.98	4.78	4.78	3.98	33.86	7	XVaA3g
8	0.62	0.62	0.49	0.37	0.49	0.99	0.12	–	2.36	29.08	3.59	4.38	4.38	3.59	33.86	8	XVaA3h
9	1.62	1.62	1.50	1.63	1.24	1.24	1.49	1.37	–	28.85	3.95	5.53	5.53	3.95	32.81	9	XVaA6
10	16.81	16.97	16.97	17.04	17.54	17.78	17.53	17.69	17.96	–	30.80	31.20	31.20	30.80	23.83	10	XVbA2G1
11	2.53	2.53	2.40	2.28	2.13	2.14	2.39	2.26	2.25	17.62	–	1.57	1.57	0.39	35.60	11	XVcA2G1
12	3.18	3.18	3.05	2.93	2.52	2.53	2.78	2.65	2.90	17.93	0.86	–	0	1.96	36.00	12	XVcA2G1a
13	3.31	3.31	3.18	3.06	2.65	2.66	2.91	2.78	3.03	18.10	0.99	0.12	–	1.96	36.00	13	XVcA2G1b
14	2.66	2.66	2.53	2.41	2.13	2.14	2.39	2.26	2.25	17.79	0.12	0.86	0.99	–	35.60	14	**XVcA2G1c**
15	19.55	19.57	19.39	19.83	20.69	20.60	20.32	20.49	19.71	12.61	21.29	21.97	22.16	21.47	–	15	XVdA3
	1	2	3	4	5	6	7	8	9	10	11	12	13	14	15		

The bolded subtype was obtained in the present study.

### Genotyping and Subtyping of *G. duodenalis*

Sequence analysis of the SSU rRNA gene identified two *G. duodenalis* assemblages: A (n = 6) and B (n = 13). All assemblage A isolates were identical to each other, and so were assemblage B isolates, which had 100% similarity with two horse-derived isolates (MN174121 and MN174122), respectively.

At the tpi locus, only two isolates of assemblage B were successfully amplified and sequenced, with two different tpi gene sequences being obtained. Due to no clear subgrouping within assemblage B, both of them were named as sub-assemblage B-I (MW011715) and B-II (MW011716) for convenient description, respectively (**Table 1**). Sub-assemblage B-I had one-base difference compared to the JX994251 and KM977638 sequences from a human and a chinchilla, respectively. Sub-assemblage B-II had 100% similarity with those isolates from a human (JX994245) and animals—non-human primates (MK982533), a bos indicus (MF459680), a dog (LC437486), a cat (LC341576), a goat (MF095053), a pig (MH644772), a rabbit (MH475909), a fox (KY304077), an orangutan (KR011753), a chinchilla (KF843914), and an anteater (GU797247).

At the bg locus, only three *G. duodenalis* isolates were successfully amplified and sequenced. The assemblage A isolate was identified as sub-assemblage AII, which was identical to those *G. duodenalis* isolates from a human (MN844143), a sheep (MK452883), and a cattle (MK452836). Two assemblage B isolates were identical to each other, and shared 100% similarity with a human-derived *G. duodenalis* isolate (MK982542).

## Discussion

To the best of our knowledge, the present study is the first report of the prevalence and genetic characterization of *Cryptosporidium* spp. and *G. duodenalis* by molecular techniques in Wa people in Myanmar. *Cryptosporidium* spp. was detected in children (1.03%, 1/97) and adults (5.88%, 2/34), while *G. duodenalis* in children (12.37%, 12/97) and teenagers (17.07%, 7/41). However, in two studies conducted in Myanmar, a prevalence of 3.4% was found in detection of either *Cryptosporidium* spp. in infants or *G. duodenalis* in schoolchildren and guardians by microscopy (Aye et al., 1994; Kim et al., 2016). The difference in prevalence may be related to detection methods employed. PCR-based molecular techniques are demonstrated to be more sensitive than conventional microscopy, such as *Cryptosporidium* spp. prevalence in sheep in Australia (26.25% versus 2.6%) (Ryan et al., 2005) and in the US (50.8% versus 20.6%) (Santín et al., 2007); *G. duodenalis* prevalence in dogs in India (20.0% versus 3.0%) (Traub et al., 2004) and in Italy (20.5% versus 11.0%) (Scaramozzino et al., 2009). Besides that, the prevalence may be related to population specimens collected and their clinical features. Children are reported to have a statistically significantly higher prevalence than adults: such as 2.56% versus 1.89% for *Cryptosporidium* spp. in China (Liu et al., 2020); 53.2% versus 22.2% for *G. duodenalis* in Uganda (Johnston et al., 2010). Meanwhile, some studies reported significant difference in prevalence of both parasites between diarrheal and non-diarrheal children, such as 16.3% versus 3.1% for *Cryptosporidium* spp. in Tanzania (Tellevik et al., 2015); 20.5% versus 8.0% for *G. dudenalis* in Ethiopia (Feleke et al., 2018). However, in the present study, *G. duodenalis* was more prevalent in non-diarrheal cases than in diarrheal cases (14.08% versus 7.69% for children; 18.52% versus 14.29% for teenagers), and all three *Cryptosporidium*-positive cases were from non-diarrheal individuals. Similar results are also reported in some previous studies, such as *Cryptosporidium* spp. in Peru and *G. duodenalis* in Ethiopia (Cama et al., 2008; Tellevik et al., 2015). Even so, the two parasites can also lead to growth and development retardation of asymptomatic children (Prado et al., 2005; Checkley et al., 2015), thus constituting a serious public health problem in this population. The prevalences are complicated and difficult to compare due to differences in the detection methods employed, the size of specimens analyzed, the populations investigated, and the health status of individuals.

*C. parvum* and *C. hominis* are recognized as the main causative agents (> 90%) in reported human cases of *Cryptosporidium* infection (Squire and Ryan, 2017; Liu et al., 2020). However, in the present study, two unusual *Cryptosporidium* species were identified: *C. andersoni* (n = 2) and *C. viatorum* (n = 1). Since the first report of *C. andersoni* in humans in 2001, there have been 144 human cases of cryptosporidiosis attributed to *C. andersoni*, composed of 141 diarrheal cases from India (n = 78) (Hussain et al., 2017), China (n = 59) (Liu et al., 2014; Jiang et al., 2014; Su et al., 2017), the UK (n = 3) (Leoni et al., 2006) and Malawi (n = 1) (Morse et al., 2007), and three cases having no information on clinical symptoms from Australia (Waldron et al., 2011), Iran (Agholi et al., 2013), and France (Guyot et al., 2001). *C. viatorum* was first identified in 10 travellers with gastrointestinal symptoms returning to the UK from the Indian subcontinent in 2012 (Elwin et al., 2012). The name was chosen to underscore its link to foreign travel. To date, 37 human cases of cryptosporidiosis including the present case caused by *C. viatorum* have been identified in nine countries: Australia (n = 1) (Braima et al., 2019), China (n = 1) (Xu et al., 2020), Colombia (n = 1) (Sánchez et al., 2017), Ethiopia (n = 12) (Adamu et al., 2014; Stensvold et al., 2015; de Lucio et al., 2016), India (n = 2) (Khalil et al., 2017; Khalil et al., 2018), Myanmar (n = 1), Nigeria (n = 2) (Ayinmode et al., 2014; Ukwah et al., 2017), Sweden (n = 3) (Insulander et al., 2013; Stensvold et al., 2015), and the UK (n = 14) (Elwin et al., 2012; Stensvold et al., 2015). 45.95% (17/37) individuals had a history of travel abroad (Kenya, Guatemala, India, Barbados, Pakistan, Nepal and Bangladesh) (**Table 5**). Travel abroad was considered to be significantly associated with an increased risk of *Cryptosporidium* infections in the US and UK studies (Hunter et al., 2004; Roy et al., 2004). Meanwhile, it was observed that 75.68% (28/37) of human cases experienced diarrhea while 13.51% (5/37) had no diarrhea (**Table 5**). Occurrence of diarrhea and the severity of cryptosporidiosis in humans are complicated, often involving the immune status of the infected hosts, the virulence of *Cryptosporidium* species/genotypes, the infective dose of oocysts and other intestinal pathogens.

**Table 5:** Geographical distribution of *C. viatorum* subtypes identified in humans and their travel history.

Host	Country/Travel history	Case number		Subtype (n)	Reference
		Diarrhea	Non-diarrhea		
Human	Australia/NA		1^a^	XVaA3g (1)	Braima et al., 2019
	China/NA		1	XVaA3h (1)	Xu et al., 2020
	Colombia/NA		1^a^	−	Sánchez et al., 2017
	Ethiopia/NA	8	2; 2^a^	XVaA3d (9)	Adamu et al., 2014; Stensvold et al., 2015; de Lucio et al., 2016
	India/NA	2		−	Khalil et al., 2017; Khalil et al., 2018
	Myanmar/NA		1	**XVcA2G1c (1)**	This study
	Nigeria/NA	2		−	Ayinmode et al., 2014; Ukwah et al., 2017
	Sweden/Kenya; Guatemala	3		XVaA3d (1); XVaA3c (1); XVaA3b (1)	Insulander et al., 2013; Stensvold et al., 2015
	UK/India; Barbados; Pakistan; Nepal; Bangladesh	13	1	XVaA3a (9), XVaA3f (2); XVaA3d (2); XVaA3e (1)	Elwin et al., 2012; Stensvold et al., 2015
Total		28	5; 4^a^	XVaA3a (9),XVaA3b (1); XVaA3c (1);XVaA3d (12); XVaA3f (2); XVaA3e (1); XVaA3h (1); XVaA3g (1); XVcA2G1c (1)	

NA, not available.

The bars denote negative results at the locus.

The bolded subtype was obtained in the present study.

a Cases having no information on clinical symptoms.

Currently, it is unclear on the source of infection/contamination of *C. andersoni* and *C. viatorum* in the investigated areas. *C. andersoni* is actually the major species causing cattle cryptosporidiosis, especially in yearlings and adults (Wang K. et al., 2019). With the accumulation of molecular epidemiological data of *Cryptosporidium*, *C. andersoni* has also been found occasionally in other animal species, such as sheep, horses, camels, golden takins, monkeys, hamsters, and ostriches (Liu et al., 2020). In a previous molecular epidemiological study of *Cryptosporidium* in diarrheal outpatients conducted in China, 21 C*. andersoni* isolates were identical to cattle/goat-derived isolates at the SSU rRNA locus (Jiang et al., 2014). In the present study, we observed high homology (99.73% and 99.62%) of the SSU rRNA gene of two *C. andersoni* isolates with a cattle-derived isolate. *C. viatorum* was initially thought to occur exclusively in humans. However, it has also been detected in some rat species in Australia (Koehler et al., 2018) and China (Chen et al., 2019; Zhao et al., 2019). The same subtypes (XVaA3h and XVaA3g) have been identified in humans (Braima et al., 2019; Xu et al., 2020) and rats (Chen et al., 2019) (**Tables 5**, **6**). In the present study, a novel subtype (XVcA2G1c) was identified in humans for the first time, which had the largest similarity of 99.88% (one-base difference) with that (XVcA2G1) from a Bower’s white-toothed rat in China (Chen et al., 2019). These results above indicated the large potential of zoonotic transmission of *C. andersoni* and *C. viatorum*. Therefore, the true burden of human cryptosporidiosis caused by *C. andersoni* and *C. viatorum* attributed to humans and animals as well as the transmission dynamic of this disease needs to be assessed in the investigated areas by systematic molecular epidemiological surveys of humans and animals in the future.

**Table 6:** Geographical distribution of *C. viatorum* subtypes in animals.

Host	Country	Case number	Subtype (n)				Reference
			XVa	XVb	XVc	XVd	
Australian swamp rats	Australia	3		XVbA2G1b (3)			Koehler et al., 2018
Edward’s long-tailed rats	China	11			XVcA2G1a (4); XVcA2G1b (1)	XVdA3 (1)	Zhao et al., 2019
	China	4	XVaA6 (2)				Chen et al., 2019
Bower’s white-toothed rats	China	21	XVaA3g (7); XVaA3h (7)		XVcA2G1 (1)		Chen et al., 2019
Total		39	XVaA6 (2); XVaA3g (7); XVaA3h (7)	XVbA2G1b (3)	XVcA2G1a (4); XVcA2G1b (1); XVcA2G1 (1)	XVdA3 (1)	

The genotyping results showed all *G. duodenalis*-positive individuals in the investigated areas were infected with assemblages A (6/19, 31.58%) and B (13/19, 68.42%). Molecular epidemiological data indicated that assemblage B commonly had higher prevalence than assemblage A worldwide (Ryan and Cacciò, 2013), such as 68.0% (66/97) versus 29.9% (29/97) in Spain (Wang Y. et al., 2019); 66.7% (8/12) versus 33.3% (4/12) in China (Yu et al., 2019). However, an oppositive pattern of distribution of assemblages A and B has also been noticed in humans. Assemblage A showed a predominance compared to assemblage B in some studies, such as 35.9% (33/92) versus 21.7% (20/92) in Ethiopia (Damitie et al., 2018); 52.5% (31/59) versus 22.0% (13/59) in Ethiopia (Gelanew et al., 2007). Although epidemiological investigations have been conducted worldwide, the number of molecular epidemiological studies of giardiasis in humans is relatively small. To date, there is a lack of geographical structuring of the *G. duodenalis* assemblages across the globe. Difference in geographical distribution of assemblages might be associated to the socioeco-epidemiological factors of the population investigated (Sánchez et al., 2017) and methodological aspects, such as targeted genes, number of loci, primers, downstream procedures etc (Oliveira-Arbex et al., 2016).

One of six assemblage A isolates was successfully amplified and identified as sub-assemblage AII based on sequence analysis of the bg gene. Currently, there are three sub-assemblages (AI, AII and AIII) identified within assemblage A. Sub-assemblages AI and AII are commonly found in animals and humans, respectively; however, so far sub-assemblage AIII has been found only in animals, mostly in wildlife (Ryan et al., 2019). Although some studies indicated that contact with farm animals was associated with an increased risk of *G. duodenalis* infection for adults (Hoque et al., 2002; Hoque et al., 2003), genotyping and subtyping data point only to the potential role for zoonotic transmission with little epidemiological support (Xiao and Fayer, 2008). Sub-assemblage AII has ever been found in both pets (dogs) and their owners in Belgium (Claerebout et al., 2009). In India, genetically similar sub-assemblage AII isolates have been found in dogs and humans living within the same household (Traub et al., 2004). The same bg gene sequences of sub-assemblage AII were observed in human (here) and in sheep and cattle (previously). Meanwhile, at the tpi locus, one assemblage B isolate had 100% similarity with those from various animals (seen in Results). The finding of the same gene sequences of *G. duodenalis* isolates derived from humans and animals indicated the possibility of zoonotic transmission in the investigated areas. Due to the lack of data of *G. duodenalis* in local animals, the epidemiologic role of animals in the spread of giardiasis will be assessed.

## Conclusion

This is the first molecular epidemiological investigation of *Cryptosporidium* spp. and *G. duodenalis* in humans in Myanmar. In general, *G. duodenalis* was more prevalent than *Cryptosporidium* spp. in the investigated areas. High percentage of non-diarrheal individuals infected with *Cryptosporidium* spp. and *G. duodenalis* should be made aware of the importance and epidemiological significance. Two unusual *Cryptosporidium* species (*C. andersoni* and *C. viatorum*) were identified, with a novel *C. viatorum* subtype XVcA2G1c being found for the first time. DNA sequences of *Cryptosporidium* spp. and *G. duodenalis* isolates from investigated people had high similarity or even identity of animal-derived isolates, implying the potential of zoonotic transmission. Due to unclear transmission patterns and dynamics of *Cryptosporidium* spp. and *G. duodenalis*, future research effort should focus on molecular epidemiological investigations of the two parasites in humans and animals living in close contact in the investigated areas, even in whole Myanmar. These data will aid in making efficient control strategies to intervene with and prevent occurrence of both diseases.

## Data Availability Statement

The representative nucleotide sequences obtained in this study were deposited in the GenBank database under the following accession numbers: MW014313 to MW014315 (SSU rRNA) and MW014316 (gp60) for *Cryptosporidium*; MW011715 and MW011716 (tpi), MW011717 and MW011718 (bg) for *G. duodenalis*.

## Ethics Statement

The studies involving human participants were reviewed and approved by The Ethics Committee of the National Institute of Parasitic Diseases, Chinese Center for Disease Control and Prevention, China, and the Myanmar Eastern Shan State Special Region 2 Ethic Health Organization. Written informed consent to participate in this study was provided by the participants’ legal guardian/next of kin.

## Author Contributions

AL and YS designed this study. YW, BG, and HL performed the experiments. YW, BG, XL, YJ, and LY analyzed the data. JC contributed reagents/materials. YW and BG wrote the first draft of the manuscript and prepared the tables and the figures. AL and YS made the final revision. All authors contributed to the article and approved the submitted version.

## Funding

This work was supported partially by the National Science and Technology Major Program of China (No. 2018ZX10713001-004 to YS), the National Key Research and Development Program of China (No. 2017YFD0501300 to YJ), the Fifth Round of Three-Year Public Health Action Plan of Shanghai (No. GWV-10.1-XK13 to JC).

## Conflict of Interest

The authors declare that the research was conducted in the absence of any commercial or financial relationships that could be construed as a potential conflict of interest.
